# β-Glucan Induces Reactive Oxygen Species Production in Human Neutrophils to Improve the Killing of *Candida albicans* and *Candida glabrata* Isolates from Vulvovaginal Candidiasis

**DOI:** 10.1371/journal.pone.0107805

**Published:** 2014-09-17

**Authors:** Patricia de Souza Bonfim-Mendonça, Bianca Altrão Ratti, Janine da Silva Ribeiro Godoy, Melyssa Negri, Nayara Cristina Alves de Lima, Adriana Fiorini, Elaine Hatanaka, Marcia Edilaine Lopes Consolaro, Sueli de Oliveira Silva, Terezinha Inez Estivalet Svidzinski

**Affiliations:** 1 Departamento de Análises Clínicas e Biomedicina, Universidade Estadual de Maringá, Maringá, Paraná, Brasil; 2 Departamento de Ciências Básicas da Saúde, Universidade Estadual de Maringá, Maringá, Paraná, Brasil; 3 Instituto de Ciências da Atividade Física e Esporte, Universidade Cruzeiro do Sul, São Paulo, São Paulo, Brasil; University of Illinois at Chicago, United States of America

## Abstract

Vulvovaginal candidiasis (VVC) is among the most prevalent vaginal diseases. *Candida albicans* is still the most prevalent species associated with this pathology, however, the prevalence of other *Candida* species, such as *C. glabrata*, is increasing. The pathogenesis of these infections has been intensely studied, nevertheless, no consensus has been reached on the pathogenicity of VVC. In addition, inappropriate treatment or the presence of resistant strains can lead to RVVC (vulvovaginal candidiasis recurrent). Immunomodulation therapy studies have become increasingly promising, including with the β-glucans. Thus, in the present study, we evaluated microbicidal activity, phagocytosis, intracellular oxidant species production, oxygen consumption, myeloperoxidase (MPO) activity, and the release of tumor necrosis factor α (TNF-α), interleukin-8 (IL-8), IL-1β, and IL-1Ra in neutrophils previously treated or not with β-glucan. In all of the assays, human neutrophils were challenged with *C. albicans* and *C. glabrata* isolated from vulvovaginal candidiasis. β-glucan significantly increased oxidant species production, suggesting that β-glucan may be an efficient immunomodulator that triggers an increase in the microbicidal response of neutrophils for both of the species isolated from vulvovaginal candidiasis. The effects of β-glucan appeared to be mainly related to the activation of reactive oxygen species and modulation of cytokine release.

## Introduction

Vulvovaginal candidiasis (VVC) is an important public health problem that affects a large number of healthy women of child-bearing age, resulting in an estimated cost of USD$1 billion per year in the United States [1]. In Europe, *Candida* species are listed as the primary cause of vaginal infections. In the United States and Brazil, although VVC is also the most common, it is preceded some times by bacterial vaginosis [2], [3]. Approximately 75% of adult women will experience at least one episode of VVC during their lifetime, among which approximately 40–50% will experience further episodes, and 5% will develop recurrent VVC (RVVC) [4]. According to the literature, *Candida albicans* is still the species that is most responsible for symptomatic episodes of VVC [5], [6]. However, a significant trend toward the emergence of non-*Candida albicans Candida* (NCAC) species, such as *C. glabrata*, has been observed in recent years [4], [7].

The pathogenesis of these infections has been intensely studied over the past two decades. Recent research has presented new hypotheses to better understand this infection [4], [5], [8]. Nevertheless, no consensus has been reached on the pathogenicity of VVC or RVVC [9], [10]. Host factors, such as cellular immune deficiency, combined with pathogen virulence and its ability to effectively evade host immune defenses appear to be important. Additionally, inappropriate treatment or the presence of resistant strains can lead to RVVC [2], [4], [10]. For infections caused by NCAC species, RVVC might be closely related to inappropriate treatment. Relatively little information is available on the immune system and susceptibility to recurrent episodes [10].

Chemotherapies that seek to improve the host immune response are an alternative to control fungal infections. β-glucans are polymeric carbohydrates that have been reported to modulate inflammatory responses *in vitro* and *in vivo* [11]–[13]. The immunomodulatory effects of β-glucans are influenced by their degree of branching, polymer length, and tertiary structure, but no agreement has been reached on the basic structural requirements for biological activity, and different types of glucans have different biological effects [14], [15]. The beneficial effects of β-glucan treatment have been attributed to modulation of the immune response, such as the stimulation of phagocytosis and activation of oxidative burst, which contribute to microbicidal activity [16]. Moreover, these carbohydrates can stimulate or suppress the secretion of cytokines [11], [17]. Among the β-glucans, laminarin has been highlighted recently because its immunomodulatory properties [13], [18].

Recent research in our laboratory demonstrated that RVVC isolates of *C. albicans* induced alterations in immunological defense mechanisms, especially those related to the microbicidal activity of neutrophils (Ratti, *et al.,* unpublished data). The present study sought to determine whether host susceptibility to RVVC caused by *C. albicans* might be reduced in β-glucan-treated neutrophils. Furthermore, better understand whether the lower incidence of RVVC caused by *C. glabrata* might be related to the immune response triggered by neutrophils. Our results suggesting that β-glucan induces biochemical alterations in the microbicidal activity of neutrophils mainly through reactive oxygen species production. We also suggest that RVVC isolate caused by *C. glabrata* does not involve impairment of the microbicidal activity of neutrophils.

## Materials and Methods

### Chemicals

Dextran, Histopaque, taurine, 3,3′,5,5′-tetramethylbenzidine (TMB), phorbol 12-myristate 13-acetate (PMA), N-formyl-Met-Leu-Phe (fMLP), fluorescein isothiocyanate (FITC), dihydrorhodamine 123, catalase, RPMI-1640 medium, lipopolyssacharide (LPS) and 5–5′-dithio-bis(2-nitrobenzoic acid) (DTNB) were obtained from Sigma Chemical Co. (St. Louis, MO, USA). Sabouraud Dextrose Agar (SDA) was obtained from Difco, (Difco, Detroit, MI, USA). Hypochlorous acid (HOCl) was prepared by diluting a concentrated commercial chlorine solution and calculating its concentration using its absorption at 292 nm. PMA was dissolved in 10 mg/ml dimethylsulfoxide (DMSO). TMB solution was prepared by dissolving 10 mM TMB and 100 mM potassium iodide in 50% dimethylformamide and 50% acetic acid (400 mM). FITC was prepared by dilution with 10% DMSO in 1 ml phosphate-buffered saline (PBS). Dihydrorhodamine 123 was dissolved in 10 mg/ml DMSO. All of the experimental groups, including the controls, were tested with final DMSO concentrations of less than 1%, a concentration that was found to not affect neutrophil viability (data not shown).

### β-glucan

β-glucan derived from *Laminaria digitata* was purchased from Sigma Chemical Co. (St. Louis, MO, USA; L-9634). This glucan has the molecular form β-(1,3;1,6)-D-glucan with a molecular weight of 5.85 kDa, and it is soluble in neutral water [12]. Stock solutions of 6 mg/ml β-glucan were aseptically prepared. The concentration of β-glucan that was used in the assays was 3 mg/ml, a concentration that does not affect the viability of neutrophils and induces a neutrophil response. The test of cell viability was determined by means of trypan blue, with 95%±10 viability. This concentration was based on a dose-response curve that used 0.5, 1.0, 3.0, 5.0, and 10 mg/ml (data not shown).

### *Candida albicans* and *Candida glabrata*

A total of six clinical isolates (5 V, 7 V, 9 V, 11 V, 55 V, and 125 V) from female vaginal secretions that belonged to the archive collection of the Laboratory of Medical Mycology, Universidade Estadual of Maringa, Brazil, and two reference strains from the American Type Culture Collection (*C. albicans*, ATCC 90028; *C. glabrata*, ATCC 90030) were used. The clinical isolates were separated into groups according to symptoms presented by the patients: asymptomatic (ASS), vulvovaginal candidiasis (VVC) and recurrent vulvovaginal candidiasis (RVVC) [7]. The identity of all of the isolates was confirmed using CHROMagar *Candida* (CHROMagar, BioMerieux, Paris, France) and matrix-assisted laser-desorption/ionization time-of-flight mass spectroscopy (MALDI TOF-MS). For the MALDI TOF-MS method, the yeasts were prepared according to a previous report [19]. Measurements were performed according to a previous study [20] with a Microflex LT mass spectrometer (Bruker Daltonics) using FlexControl software (version 3.0, Bruker Daltonics).

### Growth conditions and opsonization

For each experiment, the isolates were subcultured on Sabouraund Dextrose Agar (SDA) overnight at 37°C. The cellular density was adjusted to 2×10^7^ yeasts/ml in phosphate-buffered saline (PBS) using a Neubauer chamber. Opsonization using 2.5% serum (v/v) was achieved by incubating the samples for 30 min at room temperature. Opsonized yeast was used for all of the assays.

### Neutrophils and Total Leukocytes

Neutrophils and total leukocytes were isolated from peripheral venous blood obtained from healthy volunteers by centrifugation over a Ficoll-Hypaque gradient according to [21]. The volunteers signed a consent form, this study was approved by the Committee for Ethics in Research Involving Humans at the State University of Maringa (UEM)/Paraná, Brazil (No. 293/2006). Cell concentration and viability, determined by trypan blue exclusion, were determined in a Neubauer chamber. The purity was estimated to be higher than 98%. Neutrophils (2.0×10^6^ cells/ml) and total leukocytes (2.0×10^6^ cells/ml) were suspended in RPMI1640.

### Microbicidal activity

Killing activity was monitored in the presence and absence of neutrophils, yeast, and β-glucan according to the method described by [22] with modifications. Neutrophils (2.0×10^6^ cells/ml) that were previously treated or not with 3 mg/ml β-glucan for 30 min at 37°C were activated or not with different isolates of *C. albicans* or *C. glabrata* (RVVC, VVC, and ASS) and the reference strain (2.0×10^7^ CFU/ml). These cells were maintained at 37°C with moderate shaking for 0, 30, 60, 90, and 120 min. The samples were diluted in sterile cold distilled water and mixed vigorously on a whirlmixer for 5 min to lyse the neutrophils and then diluted in sterile saline. The number of viable yeast was then calculated by spread-plating suitable diluted samples onto SDA, followed by incubation at 37°C for 24 h. The quantity of viable yeast was calculated by colony formation unit (CFU) enumeration.

### Phagocytosis assay

The phagocytosis assay for *C. albicans* or *C. glabrata* was performed using microorganisms labeled with FITC according to the method described previously [23] with some modifications. Different isolates of *C. albicans* or *C. glabrata* (RVVC, VVC, and ASS) and the reference strain (2.0×10^7^ CFU/ml) were incubated in 10 mM PBS with 5.0 mg/ml FITC for 30 min at 37°C. The neutrophils (2.0×10^6^ cells/ml) that were previously treated or not with 3 mg/ml β-glucan for 30 min at 37°C were then placed in contact with yeast for 1 h at 37°C. The negative control consisted of neutrophils alone. The fluorescence of gated neutrophils was detected using a FACSCalibur flow cytometer (Becton-Dickinson, Rutherford, NJ, USA) equipped with CellQuest software (Joseph Trotter, The Scripps Research Institute, La Jolla, CA, USA). A total of 10,000 events were acquired in the region previously established as the one that corresponded to the neutrophils. The results were recorded as the fluorescence intensity of positive cells in the sample.

### Dihydrorhodamine 123 assay

Dihydrorhodamine 123 (DHR) is normally used to detect intracellular oxidant species production by cellular systems. The non-fluorescent probe DHR is oxidized to the fluorescent product rhodamine 123 (Rh123) when it is in contact with intracellular oxidant species as described previously [24]. Total leukocytes (2.0×10^6^ cells/ml) that were previously treated or not with 3 mg/ml β-glucan for 30 min at 37°C were activated or not with different isolates of *C. albicans* or *C. glabrata* (RVVC, VVC, and ASS) and the reference strain (2.0×10^7 ^CFU/ml) or 400 nM PMA for 30 min at 37°C. After stimulation, the cells were incubated with 50 µM DHR for 30 min, washed once, and suspended in PBS. The fluorescence of gated neutrophils was detected at FL1, with 10,000 events/gate, using a FACSCalibur flow cytometer (Becton-Dickinson, Rutherford, NJ, USA). The data were analyzed using CellQuest software (Joseph Trotter, The Scripps Research Institute, La Jolla, CA, USA), and the results were recorded as fluorescence intensity and the percentage of positive cells in the sample.

### Determination of hypochlorous acid

The formation of hypochlorous acid (HOCl) was based on the formation of taurine-chloramine that results from the reaction of HOCl with taurine as described previously [25]. Neutrophils (2×10^6^ cells/ml) that were previously treated or not with 3 mg/ml β-glucan for 30 min at 37°C were activated or not with different isolates of *C. albicans* or *C. glabrata* (RVVC, VVC, and ASS) and the reference strain (2.0×10^7^ CFU/ml) in 10 mM phosphate buffer (pH 7.4) that contained 140 mM NaCl, 1 mM CaCl_2_, 0.5 mM MgCl_2_, and 1 mg/ml glucose and incubated with 15 mM taurine at 37°C under gentle agitation. The reaction was stopped after 30 min by the addition of 20 mg/ml catalase, followed by centrifugation for 10 min at 700×*g* at 24°C. The concentration of taurine-chloramine present in the supernatant was estimated by adding 50 µl of TMB solution. After 5 min, the resulting blue product was spectrophotometrically detected at 655 nm using a plate reader (WaveX5 power-Biotech USA) and related to the standard curve (constructed by adding known concentrations of HOCl) to determine the concentration of HOCl produced.

### Luminol-enhanced chemiluminescence assay for measurement of MPO release from neutrophils

Neutrophils (2×10^6^ cells/ml) that were previously treated or not with 3 mg/ml β-glucan for 30 min at 37°C were activated or not with different isolates of *C. albicans* or *C. glabrata* (RVVC, VVC, and ASS) and the reference strain (2.0×10^7^ CFU/ml) or the presence of 400 nM PMA (positive control) for 30 min at 37°C. After incubation, the cells were immersed in ice and centrifuged at 500×*g* for 10 min at 4°C to separate the supernatant from the cells. The supernatant was used to measure MPO activity. The reaction was run in PBS, 0.1 mM H_2_O_2_, and 1 mM luminol at 37°C in a final volume of 0.3 ml [26]. Luminol (5-amino-2,3-dihydro-1,4-phthalazindione) is a chemical light amplifier. Myeloperoxidase-derived metabolites are responsible for the excitation of luminol [27]. The chemiluminescent response was monitored for 20 min at 37°C in a microplate luminometer (EG&G Berthold LB96V).

### Determination of oxygen consumption by neutrophils

Oxygen consumption by neutrophils was polarographically measured at 37°C using a Clark-type electrode positioned in a closed Plexiglas chamber. Neutrophils were placed in the oxygen electrode vessel with 2 ml of PBS solution. To estimate oxygen consumption, neutrophils (2×10^6^ cells/ml) that were previously treated or not with 3 mg/ml β-glucan for 30 min at 37°C were activated or not with different isolates of *C. albicans* or *C. glabrata* (RVVC, VVC, and ASS) and the reference strain (2.0×10^7^ CFU/ml) and 400 nM PMA (positive control) for 30 min. Oxygen uptake was monitored for 5–10 min. Oxygen uptake rates were calculated from the polarographic records using an initial concentration of dissolved oxygen of 190 µM at 37°C [28].

### Cytokine assay

Neutrophils (2×10^6^ cells/ml) that were previously treated or not with 3 mg/ml β-glucan for 30 min at 37°C and activated or not with different isolates of *C. albicans* or *C. glabrata* (RVVC, VVC, and ASS) and the reference strain (2.0×10^7^ CFU/ml) or 1 µg/ml lipopolysaccharide (LPS; positive control) were cultured for 18 h at 37°C and 5% CO_2_. The supernatants obtained from the cell cultures were collected and frozen at −40°C until tumor necrosis factor α (TNF-α), interleukin-8 (IL-8), IL-1Ra, and IL-1β were determined using an enzyme-linked immunosorbent assay (ELISA; Quantikine, R&D Systems, Minneapolis, MN, USA). As expected, the amount of cytokines found in the supernatant of the cultures displayed a broad interval because of considerable individual variability in the basal release of cytokines.

### Estimation of reduced thiol level

In the thioredoxin system, thioredoxin (Trx) is reduced to a dithiol T(SH)_2_ by thioredoxin reductase (TR). The inhibition of TR decreases total reduced thiol [29]. Free thiol levels were determined using DTNB. Different isolates of *C. albicans* or *C. glabrata* (RVVC, VVC, and ASS) and the reference strain (2.0×10^7 ^CFU/ml) were centrifuged for 5 min at 8,000 g and lysed by adding 0.5 ml of lysis buffer (50 mM Tris-Cl, 150 mM NaCl, 50 mM ethylenediamine tetraacetic acid [EDTA], pH 7.2), and approximately 0.5 g of glass beads (diameter, 425–600 µm; Sigma Chemical Co.). Lysis was performed by vortexing for 3 mixing cycles of 3 min with 1 min intervals on ice. Cellular debris was removed by centrifugation, and 100 µl of the supernatant and 100 µl of 500 mM phosphate buffer (pH 7.5) were taken in each microtiter well, followed by the addition of 20 µl of 1 mM DTNB to each well. Absorbance was measured at 412 nm using a plate reader (WaveX5 power-Biotech, USA).

### Statistical analysis

The data distribution was verified using the Kolmogorov-Smirnov and Lilliefors tests. Data with a non-normal distribution are expressed as the mean ± standard deviation (SD) of at least three independent experiments. Significant differences among means were identified using analysis of variance (ANOVA) followed by the Kruskal-Wallis test and Mann-Whitney U-test. The data were analyzed using Prism 6.0 software (GraphPad, San Diego, CA, USA). Values of *p*≤0.05 were considered statistically significant.

## Results

### β-glucan increases microbicidal activity by neutrophils activated by different isolates of *C. albicans* and *C. glabrata* (RVVC, VVC, and ASS) and the reference strain

**Figure 1:**
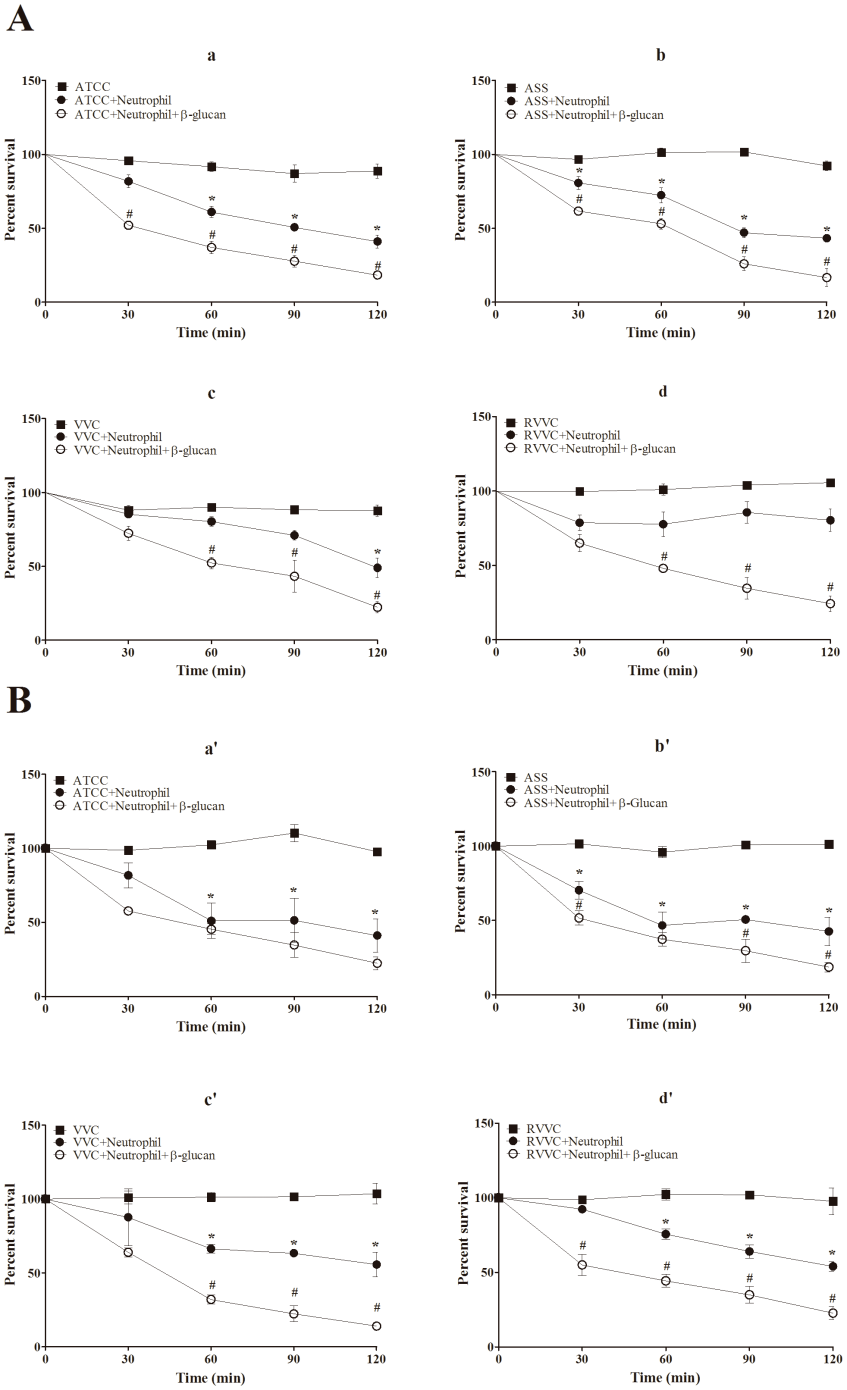
Microbicidal activity of β-glucan-treated neutrophils activated by different isolates of *C. albicans* and *C. glabrata*. Neutrophils (2.0×10^6^ cells/ml) were previously treated or not with 3 mg/ml β-glucan and incubated with the reference strain and different isolates of (A) *C. albicans* and (B) *C. glabrata* (RVVC, VVC, and ASS; 2.0×10^7^ CFU/ml) at 37°C for different times (0, 30, 60, 90, and 120 min). The quantity of viable yeast was estimated by plating the samples in Sabouraund Dextrose Agar (SDA) at 37°C for 24 h. The data are expressed as the mean ± SD of three separate experiments. * *p*≤0.05, significant difference compared with the control group (yeast alone); **^#^** *p*≤0.05, significant difference compared with untreated and activated neutrophils.

Our previous work demonstrated that the resistance of RVVC isolates caused by *C. albicans* involves the detoxification of oxidant species produced by neutrophils through TR activity, an antioxidant enzyme in fungi. Thus, the killing of the RVVC isolates was markedly impaired by the microbicidal response of neutrophils. Our first step was to evaluate the microbicidal activity of neutrophils that were previously treated with β-glucan and activated by the RVVC isolate compared with VVC and ASS isolates and the reference strain of *C. albicans* and *C. glabrata*. Fig. 1A shows that the microbicidal activity of β-glucan-treated neutrophils activated with all of the isolates of *C. albicans* increased significantly (>30%) after 60 min incubation compared with the activated and untreated neutrophil group. Importantly, this increase was time-dependent, and β-glucan-treated neutrophils killed 70% of the RVVC isolate after 120 min incubation (Fig. 1Ad). For *C. glabrata*, all of the isolates were able to induce the microbicidal activity of untreated neutrophils after 60 min incubation (Fig. 1B). This microbicidal activity was significantly higher after 60 min for the reference strain and ASS isolates (approximately 57% yeast killing) followed by the VVC and RVVC isolates (approximately 45% yeast killing) compared with the yeast alone (Fig. 1Ba’-d’). After treating the neutrophils with β-glucan, microbicidal activity significantly increased for all of the isolates, with the exception of the reference strain. Interestingly, β-glucan-treated neutrophils induced the death of more than 60% of the VVC and RVVC isolates compared with the activated and untreated neutrophil group.

### β-glucan increases the phagocytosis activity by neutrophils activated by different isolates of *C. albicans* and *C. glabrata* (RVVC, VVC, and ASS) and the reference strain

**Figure 2:**
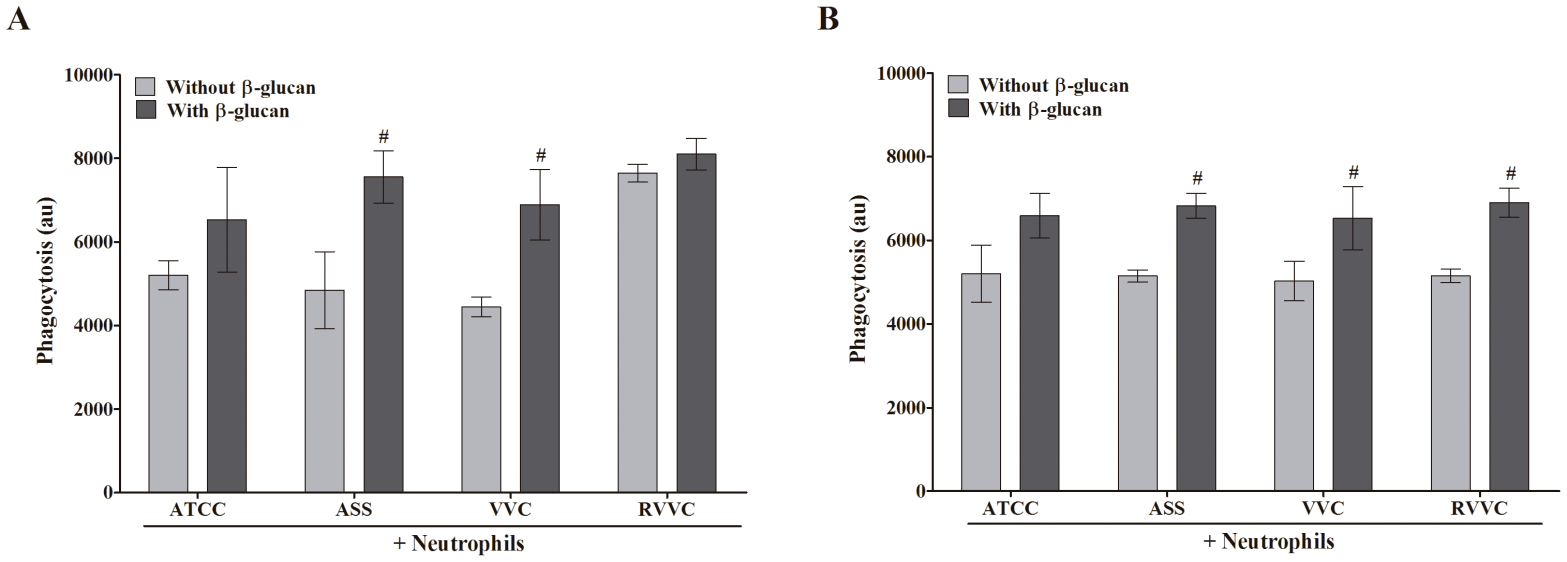
Phagocytosis activity of β-glucan-treated neutrophils activated by different isolates of *C. albicans* and *C. glabrata*. Neutrophils (2.0×10^6^ cells/ml) were previously treated or not with 3 mg/ml β-glucan and incubated for 1 h at 37°C with the reference strain and different isolates of (A) *C. albicans* and (B) *C. glabrata* (RVVC, VVC, and ASS; 2.0×10^7^ CFU/ml) labeled with FITC. Phagocytosis was determined by flow cytometry, and the results are expressed as the mean fluorescence (in arbitrary units [au]) ± SD of three independent experiments. **^#^** *p*≤0.05, significant difference compared with untreated and activated neutrophils.

Based on the effect of β-glucan on the microbicidal activity of neutrophils, we evaluated whether this carbohydrate modulates the phagocytosis of neutrophils that were activated by the RVVC isolate compared with the VVC and ASS isolates and reference strain of *C. albicans* and *C. glabrata*. All of the isolates of *C. albicans* induced neutrophil phagocytosis, with a marked rate of phagocytosis for the RVVC isolate (76%; Fig. 2A). After treating the neutrophils with β-glucan, the rate of phagocytosis increased for all of the isolates, with a significant increase for the ASS and VVC isolates (35%) compared with the activated and untreated neutrophil group (Fig. 2A). Additionally, all of the *C. glabrata* isolates induced high neutrophil phagocytosis (>50%; Fig. 2B). After treating the neutrophils with β-glucan, the rate of phagocytosis for all of the *C. glabrata* isolates increased by approximately 20% compared with the activated and untreated neutrophil group.

### β-glucan increases intracellular oxidant species production by neutrophils stimulated with different isolates of *C. albicans* and *C. glabrata* (RVVC, VVC, and ASS) and the reference strain

**Figure 3:**
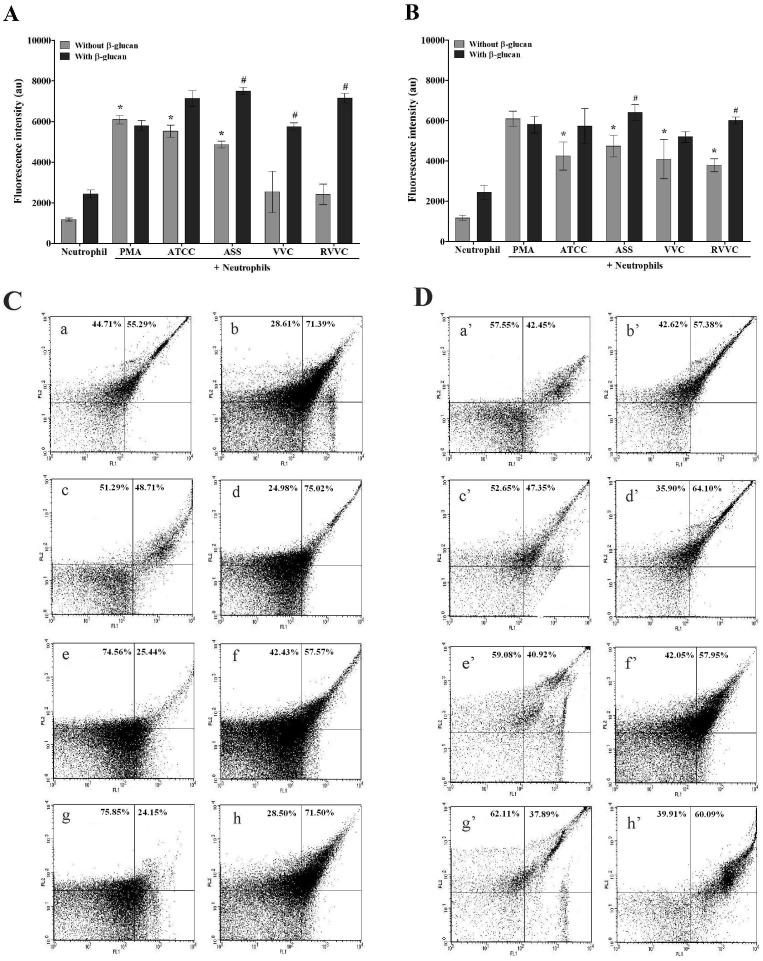
Intracellular oxidant species production by β-glucan-treated neutrophils activated by different isolates of *C. albicans* and *C. glabrata* determined by flow cytometry. Neutrophils (2.0×10^6^ cells/ml) were previously treated or not with 3 mg/ml β-glucan and incubated for 1 h with the reference strain and different isolates of (A) *C. albicans* and (B) *C. glabrata* (RVVC, VVC, and ASS; 2.0×10^7^ CFU/ml), followed by 30 min incubation with DHR. The data are expressed as the mean ± SD of at least three independent experiments. * *p*≤0.05, significant difference compared with the control group (neutrophils alone); ^#^ *p*≤0.05, significant difference compared with untreated and activated neutrophils. (C and D) Representative dot plot display of FL1 (green fluorescence) *vs*. FL2 on a logarithmic scale. (C – (a) ATCC, (c) ASS, (e) VVC,(g) RVVC) *C. albicans* with untreated neutrophils. (C – (b) ATCC,(d) ASS,(f) VVC,(h) RVVC) *C. albicans* with neutrophils previously treated with 3 mg/ml β-glucan. (D – (a’) ATCC,(c’) ASS,(e’) VVC,(g’) RVVC) *C. glabrata* with untreated neutrophils. (D – (b’) ATCC,(d’) ASS,(f’) VVC,(h’) RVVC) *C. glabrata* with neutrophils previously treated with 3 mg/ml β-glucan.

After phagocytosis, neutrophils undergo numerous signaling events that lead to the production of oxidative compounds in a complex mechanism known as oxidative burst [30]. Our early work demonstrated that the VVC and RVVC isolates of *C. albicans* did not induce significant intracellular oxidant species formation by neutrophils. Thus, we evaluated the production of total intracellular oxidant species in β-glucan-treated neutrophils that were activated by the RVVC isolate compared with the VVC and ASS isolates and reference strain of *C. albicans* and *C. glabrata*. β-glucan induced an increase in Rh123 fluorescence for all of the isolates of *C. albicans* compared with the activated and untreated neutrophil group (Fig. 3A), indicating intracellular oxidant species production by β-glucan-treated neutrophils. Interestingly, this increase in Rh123 fluorescence was significantly higher for the VVC and RVVC isolates (126% and 196%, respectively) compared with the activated and untreated neutrophil group. For *C. glabrata*, all of the isolates induced significant intracellular oxidant species production (>222%) by untreated neutrophils compared with neutrophils alone (Fig. 3B). After treating the neutrophils with β-glucan, intracellular oxidant species production significantly increased when the neutrophils were activated by the ASS and RVVC isolates (approximately 37% and 58%, respectively) compared with the activated and untreated neutrophil group. Untreated neutrophils that were activated with PMA also exhibited significant intracellular oxidant species production (418%) compared with neutrophils alone. Notably, after treating the neutrophils with β-glucan, intracellular oxidant species production increased even in inactivated neutrophils.

### β-glucan increases HOCl production by neutrophils stimulated by different isolates of *C. albicans* and *C. glabrata* (RVVC, VVC, and ASS) and the reference strain

**Figure 4:**
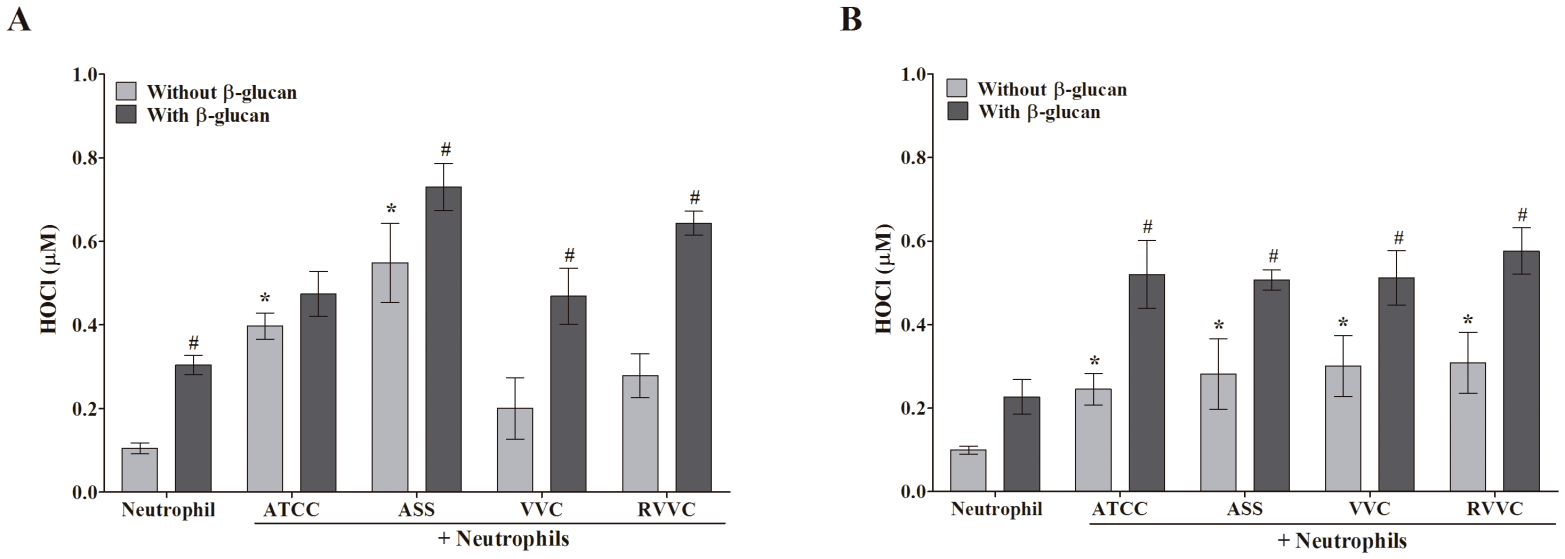
HOCl production by β-glucan-treated neutrophils activated by different isolates of *C. albicans* and *C. glabrata* determined by spectrophotometry. Neutrophils (2.0×10^6^ cells/ml) were previously treated or not with 3 mg/ml β-glucan and activated or not by the reference strain and different isolates of (A) *C. albicans* and (B) *C. glabrata* (RVVC, VVC, and ASS; 2.0×10^7^ CFU/ml) and read at 655 nm. The data are expressed as the mean ± SD of three independent experiments. * *p*≤0.05, significant difference compared with the control group (neutrophils alone); ^#^ *p*≤0.05, significant difference compared with untreated and activated neutrophils.

HOCl plays an important role in the microbicidal activity of neutrophils [31]. We studied HOCl production by β-glucan-treated neutrophils that were activated by the RVVC isolate compared with the VVC and ASS isolates and reference strain of *C. albicans* and *C. glabrata*. Untreated neutrophils that were activated by all of the isolates of *C. albicans* exhibited an increase in HOCl production. However, this increase was significant only for the ASS isolate and reference strain (at least two-fold; Fig. 4A). In neutrophils activated by all of the *C. albicans* isolates, β-glucan increased the production of HOCl after 30 min compared with the activated and untreated neutrophil group. A significant effect was observed for the ASS, VVC, and RVVC isolates, with more than a two-fold increase in HOCl production (Fig. 4A). In contrast to *C. albicans*, all of the isolates of *C. glabrata* induced a significant increase (approximately three-fold) in HOCl production by untreated neutrophils compared with neutrophils alone (Fig. 4B). After treating the neutrophils with β-glucan, HOCl production significantly increased (approximately two-fold) with all of the isolates compared with the activated and untreated neutrophil group. After treating the neutrophils with β-glucan, HOCl production increased even in inactivated neutrophils.

### β-glucan increases MPO activity by neutrophils stimulated by different isolates of *C. albicans* and *C. glabrata* (RVVC, VVC, and ASS) and the reference strain

**Figure 5:**
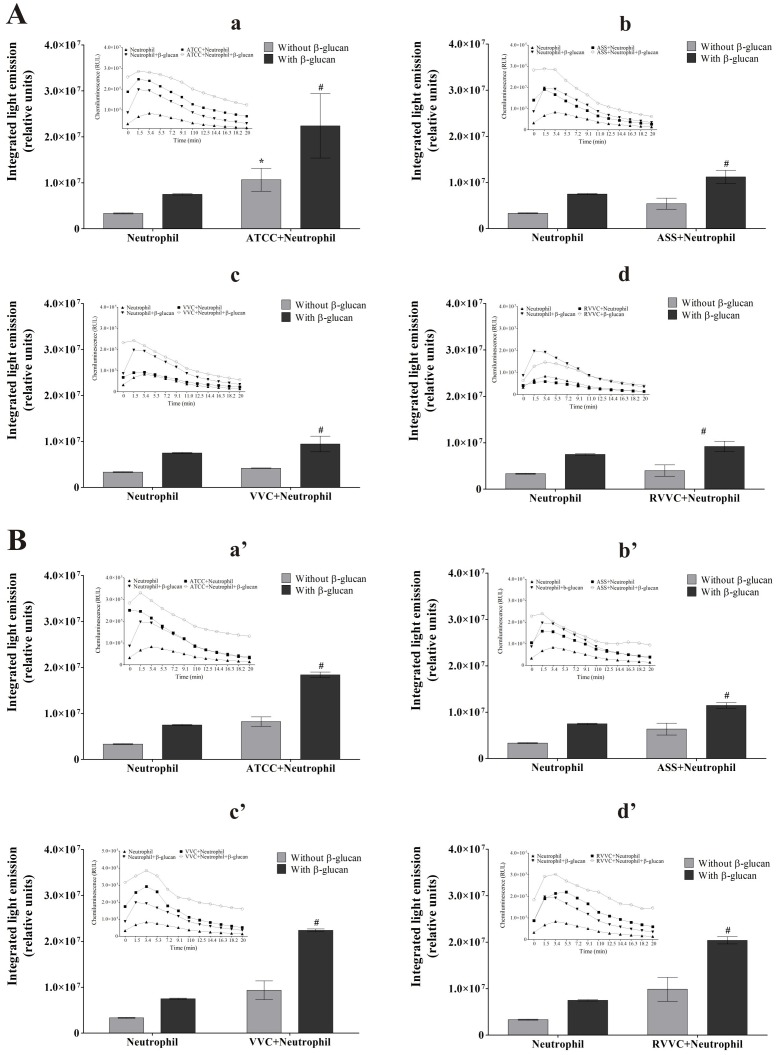
Myeloperoxidase activity of β-glucan-treated neutrophils activated by different isolates of *C. albicans* and *C. glabrata* (integrated light emission). The inset represents kinetic study of MPO activity of β-glucan-treated neutrophils after 20 minutes of incubation. Neutrophils (2.0×10^6^ cells/ml) were previously treated or not with 3 mg/ml β-glucan and incubated with the reference strain and different isolates of (A) *C. albicans* and (B) *C. glabrata* (2×10^7^ CFU/ml) for 30 min. (a,a’) ATCC. (b,b’) ASS. (c,c’) VVC. (d,d’) RVVC. After incubation, chemiluminescence was monitored for 20 min at 37°C in a microplate luminometer using luminol as a chemical light amplifier. The data are expressed as the mean ± SD of three independent experiments. *p≤0.05, significant difference compared with the control group (neutrophils alone); ^#^ *p*≤0.05, significant difference compared with untreated and activated neutrophils.

The route of HOCl generation is known to rely on MPO, a heme peroxidase enzyme that catalyzes the reaction between H_2_O_2_ and chloride [32]. Our data suggest that β-glucan activates neutrophil signaling pathways of oxidant species production, such as HOCl, leading to the removal of *C. albicans* and *C. glabrata* isolates. We evaluated the activity of MPO using luminol-enhanced chemiluminescence in both untreated neutrophils and β-glucan-treated neutrophils that were activated by the RVVC isolate compared with the VVC and ASS isolates and reference strain of *C. albicans* and *C. glabrata*. The kinetics experiment showed an increase in light emission in the first 6 min, indicating MPO activity, followed by a slight decrease in all of the groups tested (Fig. 5, inset). Fig. 5A shows that all of the *C. albicans* isolates induced an increase in MPO activity compared with neutrophils alone. Interestingly, this increase was lower for the RVVC isolate, which induced a 24% increase in MPO activity (Fig. 5Ad). Myeloperoxidase activity in β-glucan-treated neutrophils was higher for the RVVC and VVC isolates (approximately 120%) followed by the reference strain and ASS isolate (approximately 100%; Fig. 5Aa–d). For *C. glabrata*, all of the isolates induced a similar increase (>90%) in MPO activity compared with neutrophils alone (Fig. 5B). After treating the neutrophils with β-glucan, MPO activity also increased in all of the isolates compared with the activated and untreated neutrophil group (≥90%). β-glucan induced an increase in MPO activity in both inactivated neutrophils (124%) compared with neutrophils alone and neutrophils activated by all of the isolates compared with the activated and untreated neutrophil group.

### β-glucan increases oxygen consumption by neutrophils stimulated by different isolates of *C. albicans* and *C. glabrata* (RVVC, VVC, and ASS) and the reference strain

**Figure 6:**
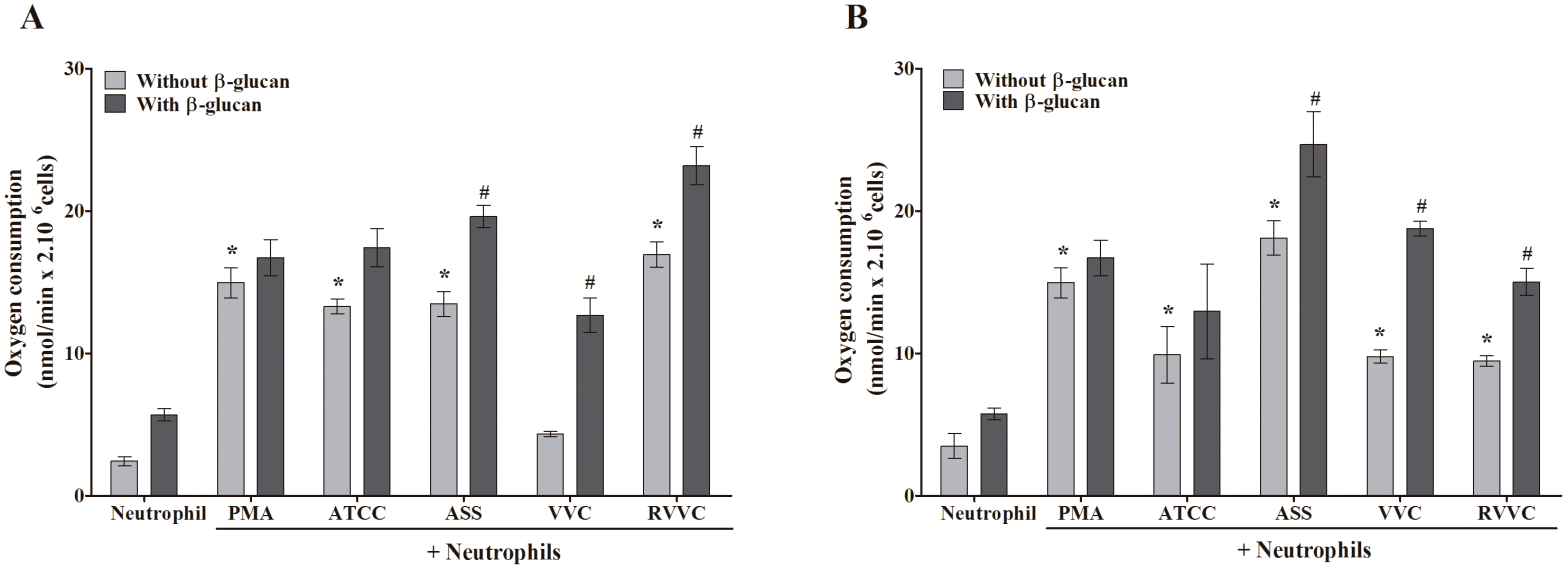
Oxygen consumption by β-glucan-treated neutrophils activated by different isolates of *C. albicans* and *C. glabrata*. Neutrophils (2.0×10^6^ cells/ml) were previously treated or not with 3 mg/ml β-glucan and activated or not by the reference strain and different isolates of (A) *C. albicans* and (B) *C. glabrata* (RVVC, VVC, and ASS; 2.0×10^7^ CFU/ml). Oxygen consumption was monitored for 5–10 min and calculated from the polarographic recordings using an initial concentration of dissolved oxygen of 190 µM at 37°C. The data are expressed as the mean ± SD of three independent experiments. *p≤0.05, significant difference compared with the control group (neutrophils alone); ^#^ *p*≤0.05, significant difference compared with untreated and activated neutrophils.

Based on the HOCl and intracellular oxidant species production results, we evaluated whether β-glucan induces oxygen consumption by neutrophils when the neutrophils were activated by the RVVC isolate compared with the VVC and ASS isolates and reference strain of *C. albicans* and *C. glabrata.* All of the *C. albicans* isolates, with the exception of the VVC isolate, significantly increased oxygen consumption by neutrophils. We showed that after treating the neutrophils with β-glucan, oxygen consumption increased with all of the *C. albicans* isolates, including the VVC isolate (Fig. 6A). This increase was significant for the ASS, VVC, and RVVC isolates (1.4-, 2.9-, and 1.4-fold, respectively). For *C. glabrata*, oxygen consumption by neutrophils was significantly higher for all of the isolates compared with neutrophils alone (Fig. 6B). This increase was more pronounced for the ASS isolate (approximately 5.2-fold), followed by the reference strain and VVC and RVVC isolates (2.8-, 2.7-, and 2.6-fold, respectively). After treating the neutrophils with β-glucan, they consumed more oxygen compared with the activated and untreated neutrophil group. This oxygen consumption was significantly higher for the ASS, VVC, and RVVC isolates (1.3- to 1.9-fold). The positive control, PMA, induced 4.2-fold higher oxygen consumption by neutrophils compared with neutrophils alone. After treating the neutrophils with β-glucan, oxygen consumption increased, even in inactivated neutrophils.

### β-glucan decreases the release of IL-8, IL-1B and TNF-a by neutrophils stimulated by different isolates of *C. albicans* and *C. glabrata* (RVVC, VVC, and ASS) and the reference strain

**Figure 7:**
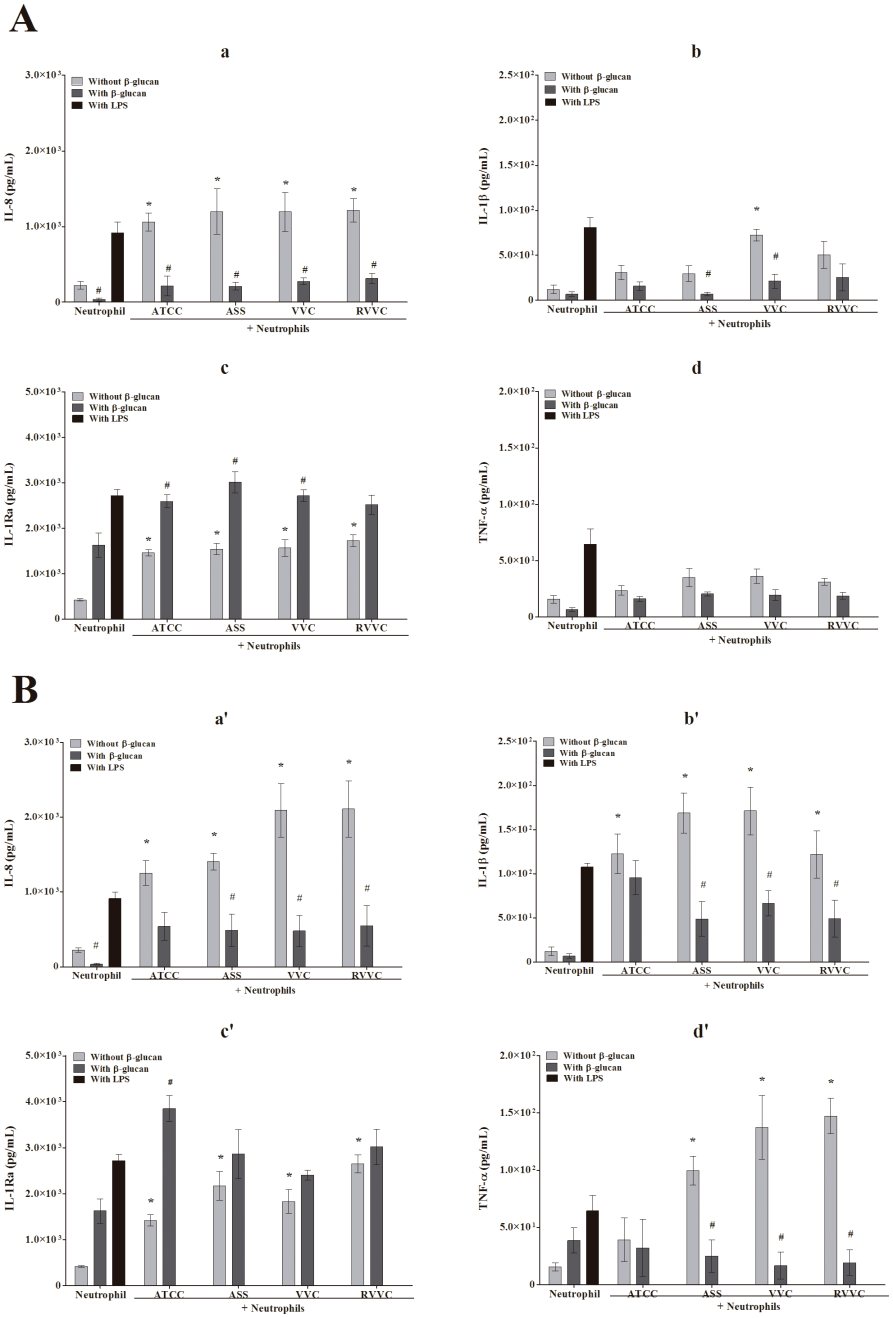
Cytokine release by β-glucan-treated neutrophils activated by different isolates of *C. albicans* and *C. glabrata*. Neutrophils (2.0×10^6^ cells/ml) were previously treated or not with 3 mg/ml β-glucan and activated or not by the reference strain and different isolates of (A) *C. albicans* and (B) *C. glabrata* (RVVC, VVC, and ASS; 2.0×10^7^ CFU/ml) and 1 µg/ml LPS and cultured for 18 h. (a, a’) IL-8. (b, b’) IL-1β. (c, c’) IL-1Ra. (d, d’) TNF-α. The data are expressed as the mean ± SD of three independent experiments. *p≤0.05, significant difference compared with the control group (neutrophils alone); ^#^ *p*≤0.05, significant difference compared with untreated and activated neutrophils.

The production of intracellular oxidant species by neutrophils is important for both microbicidal activity that leads to the oxidative damage of essential pathogenic molecules and biological signaling events that act as second messengers that regulate the production of biomolecules. We further evaluated IL-8, IL-1β, IL-1Ra, and TNF-α release in untreated neutrophils and β-glucan-treated neutrophils activated by the RVVC isolate compared with the VVC and ASS isolates and reference strain of *C. albicans* and *C. glabrata*. Fig. 7 shows that all of the isolates of *C. albicans* were able to induce the release of IL-8, IL-1β, IL-1Ra, and TNF-α compared with neutrophils alone. After treating the neutrophils with β-glucan, they exhibited a decrease in the release of TNF-α, IL-8, and IL-1β (Fig. 7Aa–c) in both inactivated and activated neutrophils. This decrease was significant for IL-8 for all of the *C. albicans* isolates (approximately 74%) and significant for IL-1β for the ASS and VVC isolates (75% and 70%, respectively). Fig. 7Ad shows that β-glucan-treated neutrophils exhibited an increase in the release of IL-1Ra in both inactivated and activated neutrophils. This increase was similar among all of the isolates (approximately 4.1-fold). For *C. glabrata*, the release of TNF-α, IL-8, and IL-1β by untreated neutrophils followed the same pattern as the one observed for *C. albicans* (Fig. 7B). However, neutrophils that were activated by the isolates of *C. glabrata* exhibited a higher level of cytokine release compared with isolates of *C. albicans.* After treating the neutrophils with β-glucan, they exhibited a significant decrease in TNF-α, IL-8, and IL-1β release for all of the isolates, with the exception of the reference strain (Fig. 7Ba–c). This decrease was similar among all of the isolates and more marked for TNF-α (approximately 8.5-fold), followed by IL-8 (4.3-fold) and IL-1β (3.5-fold). For IL-1Ra, β-glucan induced an increase in release when the isolates were activated by all of isolates of *C. glabrata*. This increase was significant for the reference strain (2.7-fold). After treating the neutrophils with β-glucan, IL-8, IL-1β, and TNF-α release decreased in inactivated neutrophils, and IL-1Ra release increased.

### Estimation of reduced thiol levels in different isolates of *C. albicans* and *C. glabrata* (RVVC, VVC, and ASS)

**Figure 8:**
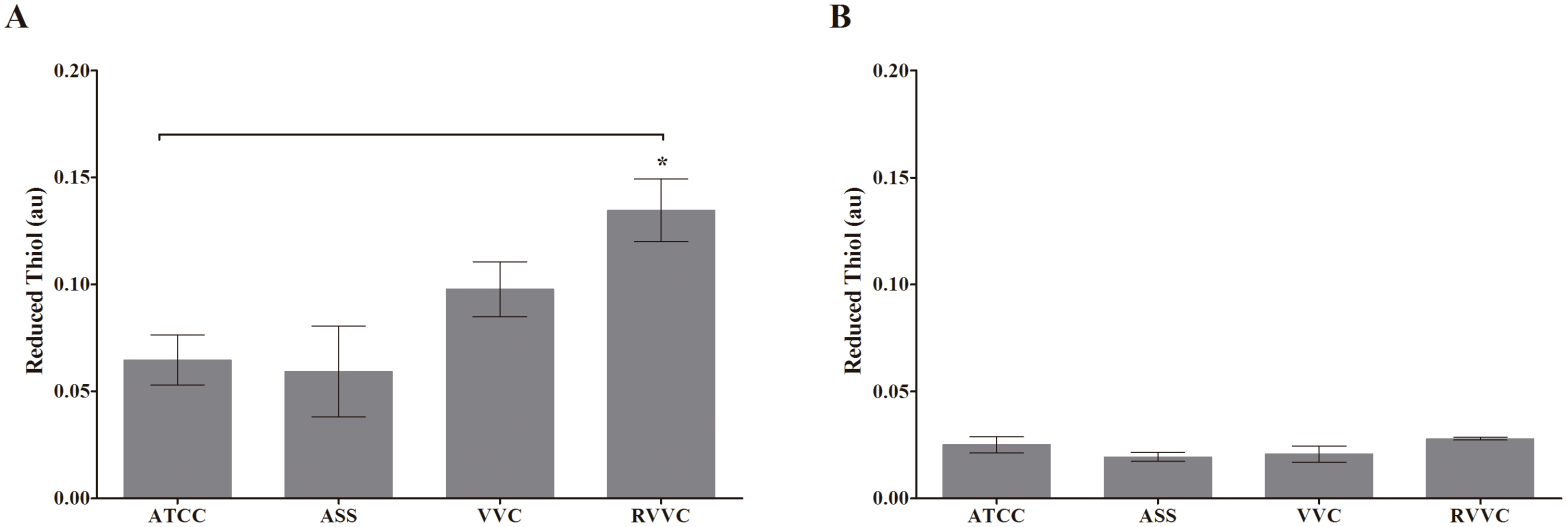
(A) Thiol levels in different isolates of *C. albicans* (ASS, VVC, and RVVC) and (B) thiol levels in different isolates of *C. glabrata* (ASS, VVC, and RVVC) incubated with DTNB. The data are expressed as the mean ± SD of three independent experiments. *p≤0.05, compared with ASS or control group (neutrophils).

The thioredoxin system, including thioredoxin (Trx) and TR, is used for oxidative stress defenses in fungi [33]. The conditions of oxidative imbalance depend on both increased oxidant species and decreased antioxidant effectiveness [34]. Our previous work (e.g. Ratti, *et al.,* unpublished data) suggested that the susceptibility of the host to the RVVC isolate of *C. albicans* is related to the activity of TR. The present data showed that *C. glabrata,* including the RVVC isolate, is sensitive to host defenses. We evaluated thiol levels in the ASS, VVC, and RVVC isolates of *C. glabrata* and reference strain. Similar to our previous work, Fig. 8A shows that thiol levels were significantly higher (approximately 39% and 55%) for the VVC and RVVC isolates of *C. albicans* compared with the reference strain. Thiol levels for *C. glabrata* were similar among the clinical isolates, with no statistically significant difference (Fig. 8B).

## Discussion

The pathogenesis of VVC caused by *Candida* species is complex and involves factors related to both fungi and the host. Depending on these factors, recurrent episodes of candidiasis might increase, making the treatment of candidiasis more challenging [2], [6]. The incidence of recurrent episodes of candidiasis is closely related to *Candida* species. To address these issues, research has sought to describe new virulence factors and fitness attributes of *Candida* species. In recent years, increased information about the mechanisms of fungal recognition by the host immune system has been reported [34], [35]. In addition to understanding how host defenses are activated, fungal pathogens are known to possess strategies to evade recognition and inhibit antifungal defenses [4], [36], [37].

In VVC episodes, neutrophils are known to be markedly recruited to the site of infection, and reactive oxygen species play a central role in host defense by killing microbes in phagocytic cells [9]. In the present study, we investigated differences in yeast-neutrophils interactions with *C. albicans* and *C. glabrata* isolates from VVC in the presence of β-glucan as an immunomodulator of the VVC response.

The incidence of *C. glabrata* ranks second in the epidemiology of VVC [4], [7], but little is known about its interactions with the host. Our results provide clear evidence that RVVC infection caused by *C. glabrata* does not appear to involve impairment of the microbicidal activity of neutrophils, in which all of the isolates were susceptible to the killing action of neutrophils. The microbicidal activity of neutrophils was activated by all of the isolates inducing significant oxygen consumption by the NADPH oxidase system, followed by high production of intracellular oxidant species, reflected by HOCl and DHR assays and also MPO activity. Interestingly, all of *C. glabrata* isolates had similarly low levels of thiol groups, reflecting TR activity. The efficient oxidative activity of neutrophils combined with the low antioxidant activity of *C. glabrata* leads to the marked removal of these isolates, revealing the susceptibility of *C. glabrata* to the action of neutrophils. Other authors reported that the lower incidence of VVC caused by *C. glabrata* might be related to the ability of this species to induce an adequate immune response [4],[38],[39]. More evidence of this was found when immunocompromised hosts developed the infection with recurrent episodes [39]. After treating the neutrophils with β-glucan, the neutrophil oxidative parameters were markedly improved, with the exception of cytokines, resulting in the greater killing of *C. glabrata* isolates by neutrophils.

In contrast to isolates of *C. glabrata*, which were equally susceptible to the action of neutrophils, *C. albicans* showed heterogeneous behavior among the clinical isolates, demonstrated mainly for VVC and RVVC isolates. For *C. albicans*, the NADPH oxidase was activated, reflected by high oxygen consumption, but MPO and HOCl production was impaired. These results suggest that high concentrations of TR in RVVC isolates caused by *C. albicans* enable them to detoxify ROS/RNS and hinder the microbicidal action of neutrophils. The VVC isolates, in contrast, had moderate TR activity, which would also be able to detoxify oxidative products. However, the low oxygen consumption in these isolates indicated possibly no activation of the NADPH oxidase system. For *C. albicans* isolates, the treatment of neutrophils with β-glucan resulted in significant phagocytosis of the ASS and VVC isolates, but the phagocytosis of the RVVC isolate remained the same as before treatment. The RVVC isolate has high virulence factors that direct the pathogenesis of recurrent episodes through the activation of an exacerbated immune response [40]. Thus, we believe that RVVC isolates boost the highest neutrophil phagocytosis response, which cannot be improved by β-glucan. β-glucan also improved the microbicidal activity of neutrophils, killing all of the isolates tested, including the RVVC isolate, which is considered resistant to the microbicidal action of neutrophils (Ratti, *et al.,* unpublished data). Based on the literature, this effect of other types of β-glucan might be related to the induction of ROS production by leukocytes [16]. Our results show that β-glucan-treated neutrophils activated by all of the isolates exhibited high ROS production, including HOCl, an important fungicidal component of oxidative metabolism in neutrophils. Thus, we believe that β-glucan induces signaling pathways that are strongly related to the activation of neutrophil oxidative burst through the NADPH oxidase system. We confirmed this action, showing that this carbohydrate induced high amounts of oxygen consumption, even when the neutrophils were activated by VVC isolates. β-glucans has also been described as an efficient immunomodulator of leukocytes that increases different biochemical processes [16]. We found that this carbohydrate might also induce the activity of reactive oxygen species-forming enzymes, reflected by an increase in MPO activity in all of the isolates tested. Interestingly, these isolates induced very low MPO activity in untreated neutrophils.

The isolates from both species were able to stimulate the production of the cytokines tested, mainly IL-8 and IL-1β. Proinflammatory cytokine release is important in the recruitment of neutrophil activation (e.g., IL-8) and stimulation of the proliferation and production of inflammatory molecules (e.g., IL-1β) [40]. The literature shows that epithelial cells release cytokines and chemokines, confirming that epithelial cells that are in contact with *Candida* spp. initiate an inflammatory response to cause tissue irritation, a common feature of CVV [40], [41]. Our results showed that *C. glabrata* was able to induce higher levels of cytokine release by neutrophils compared with *C. albicans*. β-glucan was shown to be strongly related to the oxidative metabolism of neutrophils, which induces a decrease in cytokine production compared with untreated neutrophils. These results are supported by the observation of an increase in the release of IL1-Ra by neutrophils. This interleukin is a member of the IL-1 family that binds to IL-1 receptors but does not induce any intracellular response and is characterized as a receptor antagonist [42]. The balance between IL-1β and IL-1Ra in local tissue plays an important role in the susceptibility to and severity of many diseases [40], [42]. Our results show clear evidence that β-glucan was able to maintain this balance, decreasing the release of IL-1β, likely by increasing its antagonist. A defined and balanced immunomodulatory response is crucial for protecting mucosal surfaces from coming into contact with pathogenic microorganisms [35], [41], [42]. Therefore, β-glucan serves as a modulator of the immune response, increasing ROS/RNS independently of the type of prior neutrophil activation (e.g., *C. albicans* or *C. glabrata*), improving the microbicidal activity of the host, and reducing the inflammatory response. For VVC, this response is apparently important because it enables the reduction of the classic symptoms of this pathology [40], [41].

Our results also showed that β-glucan, beyond activating signaling pathways, pre-activated dormant neutrophils, acting as a priming agent. This effect has been described for other types of β-glucan, and its action might be related to interactions with neutrophil cell surface receptors [16], [43].

Altogether, we hypothesize that β-glucan induces reactive oxygen species production at high concentrations. Reactive oxygen species are part of the microbicidal repertoire of neutrophils. More than that, ROS at very low concentration are involved in signaling events as second messenger leading to the production of essential molecules, such as cytokines. All of these functions of reactive oxygen species are very thin regulated, and the specific concentrations of ROS play an important role in this process [43].

Our analysis of untreated neutrophils indicated that the microbicidal response was activated in both *C. glabrata* and *C. albicans*. However, all of the parameters analyzed for *C. glabrata* were significantly lower compared with *C. albicans*, with the exception of cytokines. *C. glabrata* has lower virulence factors than *C. albicans*, mainly related to the ability of *C. albicans* to undergo a morphological transition from blastoconidia to hypha formation [44], [45]. In fact, the relatively low pathogenicity of *C. glabrata* compared with *C. albicans* in animal models suggests that infections caused by *C. glabrata* may not require the stringent host immune response mechanisms that are required for infections caused by *C. albicans* [46]. Additionally, the increased prevalence of *C. glabrata* infections in immunocompromised patients indicates the importance of at least a moderate host immune response. Furthermore, one of the reasons for the proliferative success of *C. glabrata* is its relatively high drug resistance, particularly toward different azole antifungals. In the last decade, *C. glabrata* emerged as a cause of mucosal and invasive fungal infections, in part because of its intrinsic or acquired resistance to azole antifungals [47].

In conclusion, as more is understood about the pathogenesis of fungal diseases from immunological, infectious disease, and microbiological perspectives, more we hope about thedirect development of future therapeutics for VVC and RVVC. Our results suggest that β-glucan is an efficient immunomodulator that triggers an increase in the microbicidal response of neutrophils that is mainly related to reactive oxygen species production for both species studied. For *C. albicans*, β-glucan decreased host susceptibility to the RVVC isolate, which has an efficient detoxification system. Additionally, β-glucan markedly increased ROS production by neutrophils that were activated by the VVC isolate, which has a low ability to induce neutrophil oxidative bursts. This effect of β-glucan is consistent with previous studies that reported that β-glucan is an immunomodulator that improves human response to infections process [12], [16]. Considering that these species, mainly *C. glabrata*, are becoming more resistant to conventional treatments, β-glucan might be used in combination with classical fungicidal drugs to induce a better host response.
